# Worldwide Increasing Incidence of Thyroid Cancer: Update on Epidemiology and Risk Factors

**DOI:** 10.1155/2013/965212

**Published:** 2013-05-07

**Authors:** Gabriella Pellegriti, Francesco Frasca, Concetto Regalbuto, Sebastiano Squatrito, Riccardo Vigneri

**Affiliations:** ^1^Endocrinology, Garibaldi-Nesima Hospital, Via Palermo, 636, 95122 Catania, Italy; ^2^Endocrinology, Department of Clinical and Molecular Biomedicine, University of Catania, Garibaldi-Nesima Hospital, Via Palermo 636, 95122 Catania, Italy; ^3^Endocrinology, Garibaldi-Nesima Hospital, and Humanitas, Catania Cancer Center, Catania, Italy

## Abstract

*Background*. In the last decades, thyroid cancer incidence has continuously and sharply increased all over the world. This review analyzes the possible reasons of this increase. *Summary*. Many experts believe that the increased incidence of thyroid cancer is apparent, because of the increased detection of small cancers in the preclinical stage. However, a true increase is also possible, as suggested by the observation that large tumors have also increased and gender differences and birth cohort effects are present. Moreover, thyroid cancer mortality, in spite of earlier diagnosis and better treatment, has not decreased but is rather increasing. Therefore, some environmental carcinogens in the industrialized lifestyle may have specifically affected the thyroid. Among potential carcinogens, the increased exposure to medical radiations is the most likely risk factor. Other factors specific for the thyroid like increased iodine intake and increased prevalence of chronic autoimmune thyroiditis cannot be excluded, while other factors like the increasing prevalence of obesity are not specific for the thyroid. *Conclusions*. The increased incidence of thyroid cancer is most likely due to a combination of an apparent increase due to more sensitive diagnostic procedures and of a true increase, a possible consequence of increased population exposure to radiation and to other still unrecognized carcinogens.

## 1. Introduction

Thyroid cancer is the most common endocrine cancer (approximately 1.0%–1.5% of all new cancers diagnosed each year in the USA) [1], and its incidence has continuously increased in the last three decades all over the world. This trend is present on every continent (Table 1) except Africa [2], where detection is possibly insufficient. The increasing incidence is indicated by the annual percent change (APC) that in the USA was 2.4% from 1980 to 1997 and 6.6% from 1997 to 2009 (both genders) (Cancer of The Thyroid-SEER Stat Fact Sheets, available at http://seer.cancer.gov/statfacts/html/thyro.html accessed on December 10, 2012). Based on recent data, thyroid cancer is the fifth most common cancer in women [3], and in Italy, it is the second most frequent cancer in women below 45 years of age [4]. Only in few countries (Norvay, Sweden) thyroid cancer incidence is decreased [2].

Genetic factors, environmental influences, and access to medical care can easily explain the high variability (up to nearly tenfold) in the thyroid cancer incidence by geographic area and ethnicity. 

Recent reports indicated a similar age-specific trends by racial/ethnic groups. Although the lowest rates of thyroid cancer are observed in blacks, the greatest rate of papillary thyroid cancer acceleration occurs in black females [5]. Male and female annual percent change was 6.3% and 7.1% for white patients, 4.3% and 8.4% for black, 4.2% and 6.7% for Hispanic and 3.4% and 6.4% for Asian/PI (Pacific Islander) patients respectively [5]. 

**Figure 1:**
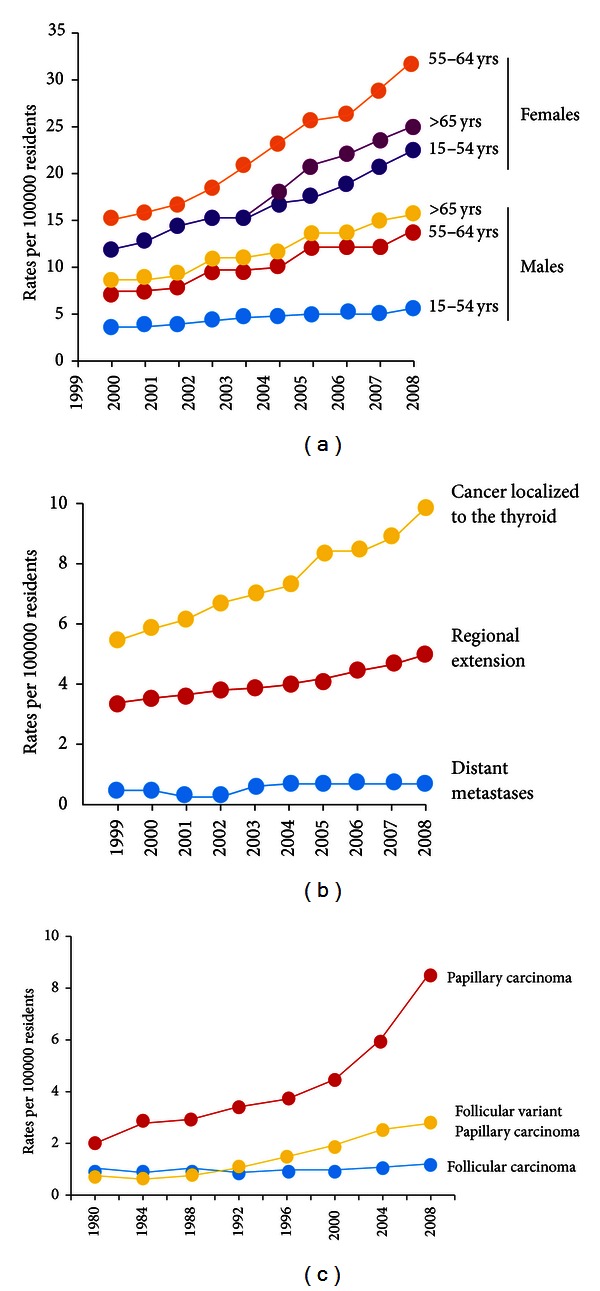
The trend in thyroid cancer incidence in the North American population from 1999 to 2008, subdivided by gender (a) and by disease stage at the time of diagnosis (b) (modified from Simard et al.) [11]. The trend in thyroid cancer incidence in the United States from 1980 to 2009 by histotype (c) (modified from Aschebrook-Kilfoy et al.) [20].

In any case, the continuously increasing rate of thyroid cancer is independent of the underlying incidence rates [2]. The increase is nearly exclusively due to increases in the incidence of the papillary histotype, with no significant change for the follicular, medullary, or anaplastic histotypes (Figure 1). The increase mainly regards small tumors, although large tumors have also increased [6, 7].

In spite of the steadily increased incidence, thyroid cancer mortality is reported stable at approximately 0.5 cases per 100,000 persons [8]. At variance with most tumors (including breast, colon-rectum, lung, and prostate cancer) whose mortality has decreased in the last two decades, thyroid cancer mortality rate is not decreased, but rather slightly increased. Indeed, the jointpoint trend reported by SEER for the period 1988–2009 (Cancer of The Thyroid-SEER Stat Fact Sheets, available at http://seer.cancer.gov/statfacts/html/thyro.html, accessed on December 10, 2012) indicates a significant increase of thyroid cancer mortality (+0.8% annual percent change, APC), primarily in males. This increase in mortality rate occurred in spite of early diagnosis and better treatment of high risk thyroid cancers. The divergence between the sharp increase in incidence and mortality, therefore, is probably less relevant than first recognized also considering that the indolent behavior of most thyroid cancers would affect mortality only many years after the increase in incidence.

Explanations for the worldwide increase of thyroid cancer incidence are controversial. Some experts believe that the increased number of new cancers is due to the increased diagnostic intensity [8, 9]. Other experts believe that a true increase, due to environment and lifestyle changes, is also a likely possibility [6, 7, 10–13]. The problem is of medical and socioeconomic relevance. Since most individuals with differentiated thyroid cancer will do well, the risk of “overdiagnosis” has been hypothesized. Overdiagnosis detects diseases that will not affect patient health and survival. Detecting these diseases not only will confer little benefit to the patient (“pseudodisease”) [14] but may also cause potential damage in terms of avoidable distress, possible adverse consequences of unnecessary treatment, and increasing economic cost. Thyroid cancer can easily fall within the condition of “overdiagnosis” because it progresses slowly, causes symptoms only when advanced, and rarely causes death.

The economic cost of overdiagnosis in a thyroid cancer patient can range from hundreds to thousands dollars, depending on the extent of the examinations performed and the complexity of the intervention and followup [15]. To prevent these costs and the inconveninences of unnecessary treatment many proposals have been recently advanced, including not submitting to cytological examination nodules ≤1.0 cm  that do not have additional risk factors (Revised American Thyroid Association Management Guidelines for Patients with Thyroid Nodules and Differentiated Thyroid Cancer, available at http://thyroidguidelines.net/revised/nodules, accessed on December 12, 2012) and the “wait and see” approach without treatment for microcarcinomas [16]. Recent studies suggest that the treatment and follow-up costs can be reduced in low-risk tumors by selective, simplified procedures [17, 18]. At present this is the most rational approach, based on the judgment of various prognostic factors that affect the patient outcome. Risk stratification, however, is based on clinical characteristics that leave a margin of uncertainty, not only for recurrent disease but also for the possibility of metastatic and deadly evolution. Although very low, this possibility provides the physician and the patient with a difficult dilemma. In fact, even when considering only microcarcinomas, extracapsular extension, lymph node metastases, and/or extrathyroid extension are found at presentation in a percentage variable from 15% to 30% [19] (Cancer of The Thyroid-SEER Stat Fact Sheets, available at http://seer.cancer.gov/statfacts/html/thyro.html accessed on December 10, 2012). Distant metastases are also present in a small percentage of cases (1%–3%). These characteristics are predictors of cancer related recurrence and mortality. We should also consider that the large majority of patients with a microcarcinoma (78%), when offered observation without surgery, opted for immediate surgery [16]. 

Therefore, a better understanding of the cause(s) of increased thyroid cancer incidence rate and improved risk stratification, using specific biological and molecular markers to accurately estimate whether a subclinical thyroid malignancy will remain stationary or will progress to an adverse outcome, is a priority to avoid overtreatment. 

## 2. Review

### 2.1. Has the Thyroid Cancer Incidence Truly Increased?

#### 2.1.1. Arguments in Favor of an Apparent Increase

The more frequent use of sensitive diagnostic procedures, including ultrasound, Doppler examination, imaging techniques like CT scan, MRI or PET scanning, and biochemical markers, has increased the detection of many types of cancer. As far as the thyroid is concerned, ultrasound and cytology examinations have identified an increasing number of small, asymptomatic thyroid cancers. Ultrasound use, in particular, has boosted the detection of small thyroid nodules that would have gone undetected in clinical practice (only 40% of thyroid nodules smaller than 1.5 cm in maximum diameter are discovered during a physical examination) [21]. The high prevalence of thyroid nodules, affecting up to 30%–50% of the population in late adulthood [21], constitutes an enormous reservoir of potential cancer lesions to be investigated. Moreover, incidental thyroid nodules (including malignant nodules) today are frequently identified after diagnostic procedures for different primary diseases. Doppler examination of the neck vessels and other imaging procedures like PET scan, for instance, have increased the detection of incidental thyroid tumors [22, 23]. Finally, the incidental discovery of preclinical thyroid tumors at pathology examination may be more frequent because of the increased use of enlarged surgical excision (total or subtotal thyroidectomy) for nonmalignant thyroid diseases [9]. In support of an increased screening effect is also the observation that thyroid cancer is positively associated with high levels of income, education, and other socioeconomic indicators of healthcare access [24]. This factor, however, should have also increased the detection of other tumors whose incidence has not increased.

The diffuse use of sensitive diagnostic procedures may have affected the incidence of cancers in the thyroid more than in other sites because thyroid cancer has an indolent progression and may remain unrecognized in the preclinical stage for years or decades, as confirmed by the observation of a high frequency (2.8%–39%) of small thyroid cancers during autopsies [25, 26]. The observation that most thyroid cancers (over 80%) are smaller than two centimeters at the time of diagnosis [8] is consistent with the possibility that increased surveillance and more sensitive diagnostic procedures are detecting cancers that were also present in the past but went undiscovered (Table 2).

#### 2.1.2. Arguments in Favor of a True Increase

When improved detection is the only cause, the increase of small, early stage tumors should be accompanied by a progressive decline of larger and more advanced tumors. The thyroid cancer increase, while most prominent for small tumors, occurred across all tumor sizes and stages, suggesting that increased detection is not the only cause [6, 10]. From 1992 to 1995, in the USA, approximately 50% of the thyroid cancer incidence increase was due to tumors ≤1.0 cm, 30% to tumors 1.1–2.0 cm, and 20% to tumors >2.0 cm [6]. In another study, the incidence rate increased for tumors <2.0 cm and >4.0 cm in size but not for tumors of medium size (2.0–4.0 cm) [7]. In Spain, from 1978 to 2001, the thyroid cancer incidence increased equally in microcarcinomas and in larger tumors [10]. Recently, an increased incidence for thyroid cancers of all stages (localized, regional, and distant staged disease) has been confirmed (Figure 1) [11]. The increased incidence of thyroid cancers of large volume and advanced stages, usually clinically apparent, can hardly be explained by increased detection.

Moreover, the thyroid cancer increase almost exclusively occurred for papillary tumors (Figure 1), while improved detection should have affected all histotypes. Recently, analysing the incidence patterns in SEER data in the United States during 1980–2009, Aschebrook-Kilfoy et al. described a modest increase in age-adjusted follicular thyroid cancer rates (Figure 1) [20].

Although improved detection can more effectively favor the identification of small, nonaggressive PTC which are the most indolent tumors, the small difference in cancer-related mortality between the papillary (approximately 7% at 10 years) and the follicular (approximately 15%) histotypes indicates that increased detection should have also affected the incidence of the follicular histotype. 

We cannot exclude, therefore, that specific carcinogens might have favored the molecular abnormalities typical of papillary cancer, a hypothesis supported by the increasing incidence of BRAF-positive papillary tumors over time [32, 33] (Table 3). 

Finally, when increased detection is the only cause, the cancer increase is expected to occur in all age and gender categories. Indeed, the age-adjusted incidence rates of thyroid cancer have increased among females more than males (158% versus 106%, resp.) with a clear birth cohort pattern, possibly reflecting changes in risk factors [34]. Although a different screening intensity according to age and gender cannot be excluded, the different secular trend in the two genders and the birth cohort effect suggest that increased detection is not the only cause of the increased incidence of thyroid cancer (Table 3). Also the age-specific trends by race do not support a detection effect as the reason for the increasing incidence.

### 2.2. Risk Factors That May Contribute to the Increased Thyroid Cancer Incidence

#### 2.2.1. Radiation

Exposure to ionizing radiation is a well-documented risk factor for cancer. The thyroid may be irradiated more than other tissues because of its position in the body and its ability to concentrate iodine. 

During the last 25 years, the individual radiation dose has doubled in the USA [35], from approximately 3 mSv/year in 1980 to 6 mSv/year in 2006. This variation is mainly due to medical diagnostic procedures [35]. Medical and dental diagnostic examinations have specifically increased thyroid exposure to X-rays [36]. CT scans, although accounting for only 15% of all radiological diagnostic procedures in the USA, provide more than 50% of the radiation dose absorbed by patients [37]. Because one-third of all CT scans are performed in the head/neck region, the thyroid is particularly exposed to radiation. Moreover, the use of iodinated contrast agents increases the radiation absorbed by the thyroid by up to 35% because iodine blocks photons, increasing the local radiation energy [38]. 

The thyroid is very radiosensitive at a young age. Children exposed to radiation frequently develop papillary thyroid cancer (PTC) as shown by the peak of thyroid cancers observed after the Chernobyl accident, when approximately 1.7 × 10^18^ Bq of ^131^I were released into the atmosphere. On that occasion, the thyroid received a dose 500- to 1000-times higher than the rest of the body, and approximately 4,000 thyroid cancer cases were reported [39]. The role of CT scans in increasing the risk of cancer in children is already documented: CT scans delivering a cumulative dose of 50–60 mGy almost triple the risk of leukaemia and brain cancer [40]. Direct evidence of CT radiation effect on the incidence of thyroid cancer in children is not available. However, risk projections can be used to estimate the potential cancer burden from CT scans on the basis of the age at scan and the type of scan. The increasing number of CT scans during childhood was hypothesized to increase the number of thyroid malignancies by up to 390 per million exposed individuals [41] and CT scans carried out in the USA in 2007 have been estimated to potentially cause about 1000 future thyroid cancers [42]. 

A recent analysis indicated that thyroid cancer risk in children exposed to head and neck radiation is inversely correlated to the age, decreasing to a nonstatistically significant level by age 15 [43]. However, a carcinogenic effect of radiation on the adult thyroid population cannot be excluded, as indicated by the increased incidence of thyroid cancer among female survivors of the atomic bomb that were older than 20 years at the time of the explosion [44]. Moreover, a recent report indicates that dental X-rays may increase thyroid cancer risk also in adults [45]. As a consequence, thyroid shielding has been recently recommended by the American Thyroid Association during diagnostic dental X-rays both in children and adults (American Thyroid Association (ATA) Issues Policy Statement on Minimizing Radiation Exposure from Medical, Dental Diagnostics, available at http://www.thyroid.org/american-thyroid-association-ata-issues-policy-statement-on-minimizing radiation-exposure-from-medical-dental-diagnostics/ accessed on December 10, 2012). 

Another specific source of thyroid irradiation is thyroid imaging with ^131^I that has been largely used for the diagnosis of thyroid diseases. Thyroid scans reached 13% of all nuclear medicine examinations in 1973 [46] and thereafter decreased to the present rate of less than 1% with ^131^I substituted by the less-dangerous ^99m^Tc [46]. The therapeutic use of ^131^I for hyperthyroidism, however, has continued or even increased, and a small increase in thyroid cancer has been observed in these adult patients [47]. Other cancer types (stomach, kidney, and breast) are also increased in patients treated with RAI for hyperthyroidism [48]. A recent meta-analysis concluded that the use of RAI for hyperthyroidism effectively increases the risk of thyroid (RR 1.99, 95% CI: 0.92–1.33), kidney (RR 1.70, 95% CI: 1.15–2.51), and stomach cancer (RR 1.11, 95% CI: 0.92–1.33) and a dose effect was also observed for the thyroid at diagnostic doses >1 Gy [49]. 

Finally, radiotherapy for head and neck malignancies is an additional source of thyroid irradiation. Indeed, in a cohort of childhood cancer survivors, 7.5% of all secondary malignancies were thyroid cancers [50]. Increased exposure to radiation, therefore, may have contributed to the increased incidence of thyroid cancer. 

#### 2.2.2. TSH Levels and Iodine Intake

Iodine deficiency causes an increase of thyroid-stimulating hormone (TSH), a major growth factor for thyroid follicular cells. Animal experiments demonstrated a clear increase of thyroid cancer after prolonged iodine deficiency leading to increased TSH. However, this effect is not demonstrated in human residents of iodine-deficient areas [51, 52]. Iodine intake is known to influence the thyroid cancer histotype distribution, rather than the overall incidence, with more follicular and fewer papillary carcinomas in iodine-deficient areas [53]. When iodine prophylaxis is introduced, average serum TSH decreases and the papillary : follicular ratio increases [54, 55]. The iodine-associated shift from a follicular to a papillary histotype may be due to the frequency of the BRAF^(V600E)^ mutation, a typical molecular alteration in PTC. BRAF-positive PTCs were significantly more frequent in Chinese regions with a high iodine intake than in control areas [56]. Although a causal relationship between iodine intake and BRAF mutation is not proven, the worldwide increase of iodine intake and the parallel increase in the prevalence of BRAF-positive PTCs [32] is in agreement with a possible role of increased iodine intake in the increased PTC incidence.

#### 2.2.3. TSH Levels and Autoimmune Thyroiditis

A major role of TSH in thyroid cancer progression is indicated by the decreased recurrence rate and improved survival in thyroid cancer patients treated with TSH-suppressive L-T4 [57]. However, a role of TSH in inducing thyroid cancer, documented in rodents, is controversial in humans. A recent study indicates that, at both univariate and multivariate analyses, the risk to have a thyroid cancer and also to have a cancer in an advanced stage is increased in patients with higher TSH serum level [58]. This correlation was confirmed in fine needle aspiration biopsies in a large series of more than 10,000 patients with thyroid nodules: the risk of malignancy was higher in patients with a higher TSH serum level [59]. Conversely, the risk of cancer was reduced in hyperthyroid patients with autonomous thyroid nodules and a low serum TSH. A similar correlation was observed also in L-T4-treated patients having serum TSH lower than untreated patients [60]. These data suggest that TSH levels, indepedently of the underlying mechanism, are positively correlated with thyroid cancer risk.

There is no evidence that serum TSH levels have increased in the population in the last decades. However, the frequency of chronic autoimmune Hashimoto's thyroiditis, the most common cause of primary hypothyroidism in the westernized world, has increased in the last two decades, paralleling the increased iodine intake [61]. Autoimmune thyroiditis might influence cancer risk not only by increasing TSH levels but also because the autoimmune process *per se*, via the production of proinflammatory cytokines and oxidative stress [62], might enhance thyroid tumorigenesis. However, the PTC frequency in patients with autoimmune thyroiditis is related to serum TSH but not to the presence of antithyroid antibodies, and when these patients are treated with LT-4 to prevent a TSH increase, the risk of thyroid cancer is no longer increased [60]. 

#### 2.2.4. Thyroid Nodules

Whether the prevalence of thyroid cancer is different in thyroid glands with a single nodule (SN) versus multinodular goiter (MNG) remains uncertain.

The prevalence of malignancy in SN has been estimated at 5% [63]. As indicated in the recent guidelines for the management of thyroid nodules patients with multiple thyroid nodules have the same risk of malignancy as those with solitary nodules [64–67]. Individual studies, however, provide cancer prevalence in patients with MNG that are lower (4.1%) [68] or higher (18%) [69]. A recent meta-analysis supported the inference that thyroid cancer is less frequent in MNG than in SN, although this finding appears to hold true mostly outside the United States, in iodine-deficient populations [70]. 

#### 2.2.5. Body Weight and Insulin Resistance

A strong correlation between obesity and cancer risk and mortality has been demonstrated for several malignancies [71]. A pooled analysis of five prospective studies indicated that also the risk of thyroid cancer is greater in obese subjects [72]. 

As obesity is a multifactorial syndrome, the contribution to cancerogenesis of each single obesity feature (type of adiposity, metabolic derangement, insulin resistance, etc.) is unclear. A recent study supports the possibility that insulin resistance and hyperinsulinemia (a typical feature of obesity) rather than metabolic derangement may be a risk factor for thyroid cancer [73]. Insulin regulates thyroid gene expression and stimulates thyrocyte proliferation, differentiation, and transformation. Insulin resistance was present in 50% of PTC patients versus 10% of matched controls [74], and BMI at the time of diagnosis was directly related to thyroid cancer risk in females [75]. Hyperinsulinemia, therefore, may be a risk factor for thyroid cancer, but its effect on the thyroid should be similar to that observed on other organs where cancer incidence has not increased [71]. The pandemia of obesity that characterizes the last decades, therefore, may have contributed to thyroid cancer increase but whether a specific effect on the thyroid is present and what is the underlying mechanism is unknown.

#### 2.2.6. Diet, Lifestyle, and Environmental Pollutants

The influences of diet, lifestyle, and pollution on thyroid cancer initiation have never been studied carefully. The evidences of a possible effect of nutrient/food or environmental pollutants on thyroid cancer are weak and not confirmed. Studies aimed at identifying cancer risk factors belonging to diet and lifestyle have provided controversial results [76] because food and drinks have a great number of different constituents (many unmeasured or highly variable) and also because dietary intake and lifestyle may significantly change in the same individual over time. 

In addition to iodine itself, dietetic factors that interfere with iodine organification and thyroid hormone synthesis, such as cruciferous vegetables, could also affect thyroid cancer risk [77]. This possibility, however, has never been demonstrated. Some industrialized food contaminants, for instance nitrates, can compete with iodine uptake by the thyroid and can behave as potential thyroid function disruptors and carcinogens. Nitrate is a frequent contaminant of drinking water in areas of intense agricultural industry and is found at high levels in some vegetables and processed food [78]. A high average nitrate level in water supplies is associated with an increased risk of thyroid cancer (highest versus lowest quartile, RR = 2.9 [1.0–8.1] [79]. 

In the last decades, the population has been more exposed to environmental pollutants like asbestos, benzene, formaldehyde, pesticides, bisphenol A (BPA), polychlorinated biphenyls (PCB), and polyhalogenated aromatic hydrocarbons (PHAHs), all compounds that may act as either genotoxic or nongenotoxic carcinogens. Particularly polybrominated diphenyl ethers (PBDEs) may induce abnormal thyroid cell proliferation, favoring a precancerous state [80]. At present, however, no causal correlation has been established between enviromental pollutants and thyroid cancer in humans. Out of 80,000 chemicals present in products on the US market, only a few hundred have been tested for carcinogenicity, and their possible combinations provide an indefinite number of potential carcinogens. It is possible that some products have a specific carcinogenic effect on the thyroid, either directly or acting as endocrine disruptors [81]. We and others [52, 82, 83] observed that the volcanic environment may be associated with an increase in thyroid cancer incidence. In the Mt. Etna volcanic area, where thyroid cancer incidence rate is more than doubled in respect to the rest of Sicily [52], only the papillary histotype is increased and both micro- and macrocarcinomas are augmented, reflecting the worldwide pattern of thyroid cancer increase. Nonanthropogenic pollutants are increased in drinking water derived from the volcanic aquifer and in the urine of the local population, confirming the increased population exposure to the pollutants, mainly heavy metals (R.V., unpublished data). A possible cause-effect relationship between these compounds and thyroid cancer is under study. If this relationship is present, it may help to unravel some of the environmental factors that favor the worldwide increase of papillary thyroid cancer.

## 3. Summary and Conclusions

The reason(s) for the worldwide increase in thyroid cancer are stil unclear. The cause is most likely multifactorial and the increased detection rate certainly represents part of the phenomenon, since small tumors are predominately increased. A true increase, however, is also likely as indicated by the increase also of large tumors, the exclusive increase of the papillary histotype only, and the differences in gender and age cohort increase. Also the lack of mortality decrease, in spite of earlier diagnosis and more efficacious treatment, supports the possibility that an increasing number thyroid cancers occurs at present. 

Increased exposure to radiation is the most likely contributing factor but also other environmental carcinogens can contribute. Although at present we have no solid evidence of a carcinogenic effect of diet and lifestyle changes and/or of environmental pollutants on the thyroid, the possibility that undiscovered environmental carcinogens might be responsible for an increased thyroid cancer incidence cannot be excluded. In particular, exposure to some chemicals during intrauterine life and early childhood, with possible epigenetic changes, might affect thyroid cell propension to mutagenesis. Further studies are warranted to investigate the potential carcinogens and their mechanism of action in order to introduce preventive measures and control the continuous increase of thyroid cancer. 

## Figures and Tables

**Table 1:** Increase of thyroid cancer incidence rate in different countries.

Country	Source	Years		Variation of incidence (APC)	
				Females	Males
Australia	[27] (*Patients aged 15–30 years*)	1982 1982 2000	2007 2000 2007	— 2.0 13.8	4.0 — —
Canada	[28] [29]	1970/72 2002	1994/96 2008	3.5* 7.3	3.2* 8.4
China (Shanghai)	[30]	1983 1983	2000 2003	— 4.9	2.6 —
Denmark	[2]	1973/1977	1998/2002	81.3%^‡^	20.0%^‡^
Finland	[2]	1973/1977	1998/2002	62.8%^‡^	29.4%^‡^
France	[31]	1983	2000	8.98	8.13
Israel-Jews	[2]	1973/1977	1998/2002	95.2%^‡^	34.6%^‡^
Italy	[4]	1991/95	2001/05	145%^‡^	127%^‡^
Japan	[2]	1973/1977	1998/2002	85.7%^‡^	52.4%^‡^
Spain	[10] (*Only PTCs*)	1978	2001	9.4^§^	2.6^§^
Switzerland	[2]	1973/1977	1998/2002	85.7%^‡^	5.3%^‡^
UK	http://info.cancerresearchuk.org/cancerstats/	1993	2008	2.3	0.6
USA	[12] http://seer.cancer.gov/statfacts/html/thyro.html	1998 1997	2005 2009	7.0 7.0	6.3 —

APC: annual percent change.

*Average annual percent increase.

^‡^Percent temporal change (% increase) in the indicated period.

^§^Incidence increase in the indicated period.

**Table 2:** Thyroid cancer incidence is increasing worldwide: possible reasons.

(A) The increase is apparent (not more cancers but more detection)	
(i) Widespread diffusion of advanced medical procedures *(ultrasounds and fine needle aspiration biopsy) *	
(ii) The increased incidence concerns mainly microcarcinomas	
(iii) Increased detection of “incidental,” microcarcinomas because	
(1) total thyroidectomies for benign lesions are more frequent	
(2) pathological examinations are more detailed	
(3) incidental discovery of nodules at diagnostic examination for other diseases is frequent	
(iv) High frequency of undiagnosed, asyntomatic small thyroid cancers at autopsy	
(v) Improved accuracy of cancer registration	
(B) The increase is true (more cancers because of changes in the risk factors)	
(i) Large tumors are also increased	
(ii) The incidence of large size and advanced stage cancers is not decreased, as expected when early diagnosis is more frequent	
(iii) Only the papillary histotype of thyroid cancer is increased	
(iv) Increased incidence is not proportionally distributed for age and gender *(secular trend is greater for females and a birth cohort pattern is present) *	
(v) Improved accuracy of cancer registration should have produced similar effects also for other tumors	
(vi) Mortality rate	
(1) stable mortality rate may result from early diagnosis and better treatment counteracting the effect of the increased cancer number	
(2) thyroid cancer progression is very slow and increased incidence would affect mortality only after decades	
(3) recent data indicate that mortality is increasing, specially in males	

**Table 3:** Potential carcinogenic factors thyroid cancer.

	Factor	Source

Exogenous	X-rays	Medical imaging (dental X-ray and CT scans)
	^ 131^I	Nuclear medicine procedures
	Iodine	Diet, iodine prophylaxis, BRAF^V600E^ (?)
	Nitrate	Water and diet
	Westernized lifestyle and environmental	Undiscovered carcinogens
	pollutants	Bisphenol A (BPA), polychlorinated biphenyls (PCB), polybrominated diphenyl ethers (PBDEs)

	Factor	Mechanism

Endogenous	TSH	Thyroid growth stimulation
	Autoimmune	increased TSH and oxidative stress
	Thyroiditis	
	Obesity and insulin resistance	Hyperinsulinemia promotes cancer, but this factor is not specific for the thyroid
